# An experimental study of the effect of pre-operative administration of cilostazol on random skin flap survival in rats: double blinded randomized controlled trial

**DOI:** 10.1186/s13022-015-0011-4

**Published:** 2015-04-29

**Authors:** Chairat Burusapat, Janjira Paengnoi, Kantang Satayasoontorn

**Affiliations:** Division of Plastic and Reconstructive Surgery, Department of Surgery, Phramongkutklao Hospital and Phramongkutklao College of Medicine, 315 Ratchawithi Road, Thung Phayathai, Ratchathewi, Bangkok, 10400 Thailand; Army Institute of Pathology, Bangkok, Thailand

**Keywords:** Cilostazol, Random skin flap in rats, Flap survival

## Abstract

**Background:**

Insufficient arterial blood flow is the one cause of flap necrosis. Cilostazol is an inhibitor of phosphodiesterase III and increases cyclic AMP level in vascular smooth muscle cell causing vasodilation. Therefore, effect of cilostazol is expected to improve the viability of the flap.

**Methods:**

Double blinded randomized controlled trial was conducted. The study was to compare the survival of dorsal rat flaps between preoperative cilostazol supplemented diet and regular diet. The flap survival area was measured using PixArea Image software on post operative day 1,3,5 and 7. Fluorescein injection was performed to evaluate the exactly area of flap survival on postoperative day 7 and morphology of arterioles and venules were examined by histopathologic examination.

**Results:**

A statistical significance was found in the percentage of area of flap survival between cilostazol supplemented diet and control group on postoperative day 3, 5 and 7 (p < 0.05). Fluorescein injection showed the higher area of flap survival in cilostazol group than the control group (p < 0.05). Histopathologic examination showed dilation of vessels in the cilostazol group.

**Conclusion:**

Preoperative cilostazol in rats can enhance skin flap survival.

**Electronic supplementary material:**

The online version of this article (doi:10.1186/s13022-015-0011-4) contains supplementary material, which is available to authorized users.

## Background

Reconstruction of tissue defect by flap coverage is widely used in the field of plastic surgery. Despite more understanding of the mechanism of flap necrosis and the advances in surgical technique, necrosis of flap remains a significant problem surgery. Flap necrosis is demonstrated by insufficient arterial flow, inadequate venous drainage or combination of both [1,2]. Many techniques have been introduced to alleviate this problem. Delay flap procedure increase the viability, however, disadvantage of this technique is requiring second operations [3-7]. Many pharmacological agents [8] have been introduced to improved ischemia of flap in experimental studies, however, clinical outcome are still controversial. These agents include sympatholytics, vasodilators, calcium channel blockers, anticoagulants [9,10], volume expander agent [11], prostaglandin inhibitors [12], and botulinum toxin A [13,14].

Cilostazol is a selective inhibitor of phosphodiesterase type III, by which it increases intracellular cyclic AMP (cAMP) and also raises the vascular smooth muscle cell’s cAMP level causing vasodilation. It is an antithrombotic that also reversibly inhibits platelet aggregation and causes arterial vasodilation. Cilostazol was approved for use in England by the National Institute for Clinical Excellence (NICE) and has been licensed in the United States since 1999, by the Food and Drug Administration (FDA). It is benefit to treat patients suffering from intermittent claudication without rest pain and no peripheral tissue necrosis as it improves pain-free walking distances [15,16]. Therefore, effect of cilostazol to the vascular smooth muscle cell that causing vasodilation is expected to increase the survival of flap. However, the effect of cilostazol on the skin flap survival has not been established.

Authors have hypothesized that pre-operative administration of cilostazol will be improved the viability of the flap. The objective is to study the efficacy of pre-operative administration of cilostazol in survival of random cutaneous flap in rat model.

## Methods

This study was approved by the Animal Care Committee of Phramongkutklao Hospital and Ethic Committee. Thirty male Sprague–Dawley rats weighing between 250 and 350 gm were used in this study and cared for under the National Research Council’s guidelines for the care and use of laboratory animals. The rats were housed in a temperature-controlled room (25 ± 2°C) on a 12 hours light: 12 hours dark cycle with free access to food and water.

**Figure 1:**
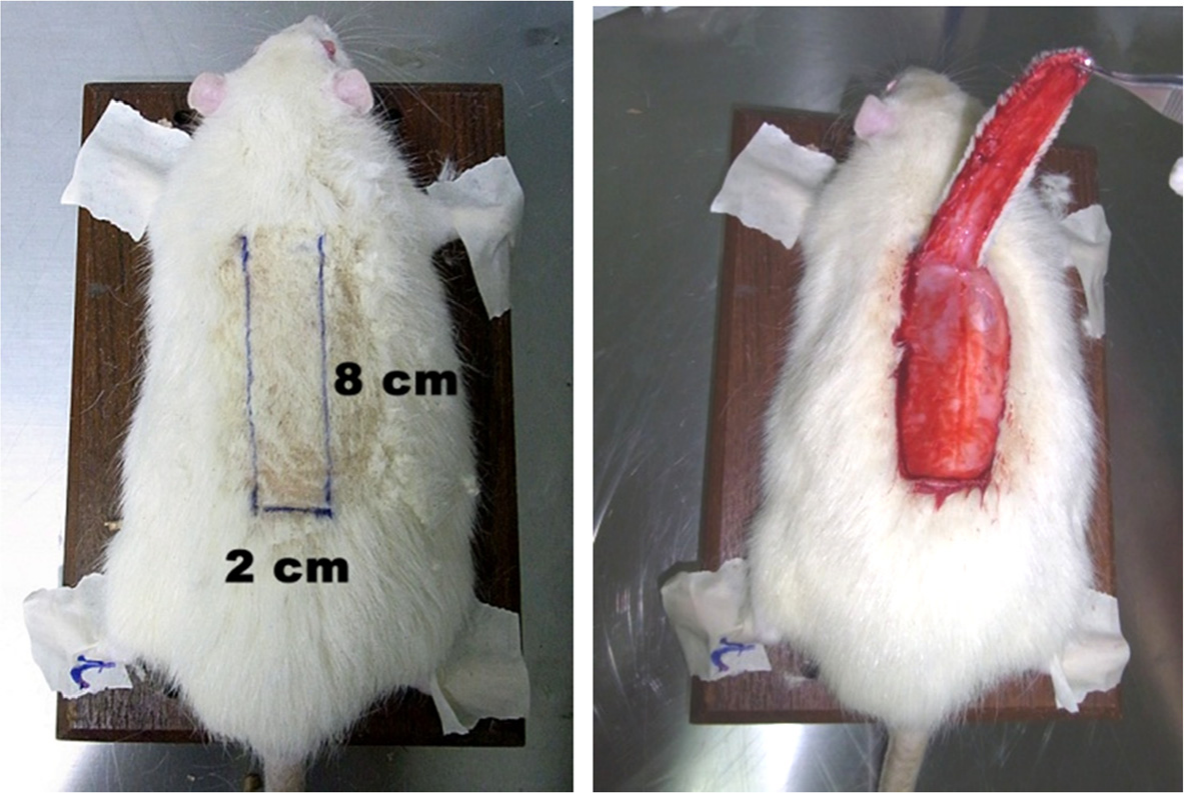
A 2X8 cm^2^ cephalic based rectangular dorsal skin flap was designed on the rats and then elevated.

Rats were randomly divided by the first surgeon into two groups (15 rats in each group). First group was given with cilostazol supplemented diet (cilostazol group) (40 mg/kg/day) every day for 7 days and the second group was the control group (regular diet). Body weight was not significantly different among groups during the experiment. All rats were performed under general anesthesia induced by intraperitoneal injections of a xylazine 2–4 mg/kg and zolitil 30 – 40 mg/kg mixtured by second surgeon (blinded about the rat groups). Following induction of general anesthesia, the skin of the dorsal trunk was shaved and then prepared with Betadine solution, and rats were placed in a prone position. A 2X8 cm^2^ cephalic based rectangular dorsal cutaneous flap was designed and elevated with base of flap at the lower margin of the scapula (Figure 1). A silicone sheath was placed under the flap to block the new blood supply from the bed, then the flap was returned to its original position and carefully sutured into place using 4/0 Nylon suture. The rats were returned to individual cages.

Flap survival was checked on postoperative day 1, 3, 5 and 7 by second surgeon. Betadine solution was cleaned on the flap by normal saline solution before taking a photograph. A digital photograph was taken 30 cm. from the rats and the PixArea Image Program (version 1.03 (2008) software, Finland was used. Using the program, the length of the image was converted to the actual length, and the surface area of flap survival was calculated. Discoloration of the flap and dry eschar formation were regarded as gross criteria of necrosis. Flap survival area was calculated by the percentage of viable surface to the total surface area by the second surgeon.

### Fluorescein testing

Fluorescein testing was performed to demonstrate the exactly survival area of the flap. Seventh day after the flap elevation, all rats were reanesthetized and 0.3 ml of 10% sodium fluorescein was administrated intravenous. Twenty minutes after the fluorescein injection, perfusion at the inner surface of flap was demonstrated under a Blacklight lamp in a darkened room. A digital photograph was taken 30 cm. from the rats. The survival area was defined by demonstration of the fluorescein appearance. The flap survival was calculated by the percentage of viable surface to the total surface area by the second surgeon and compared between groups by the first surgeon.

### Histopathological evaluation

Tissue samples were taken at 1 and 4 cm. from the base of flap and preserved in 10% formalin solution. Sampling tissues were embedded in paraffin blocks and stained with haematoxylin and eosin for histopathological examination.

### Statistical analysis

The difference in the mean area of survival and mean percentage of flap survival between the two individual groups were analyzed using the Mann–Whitney U-test. Probabilities of less than 0.05 were accepted as statistical significant.

## Results

**Figure 2:**
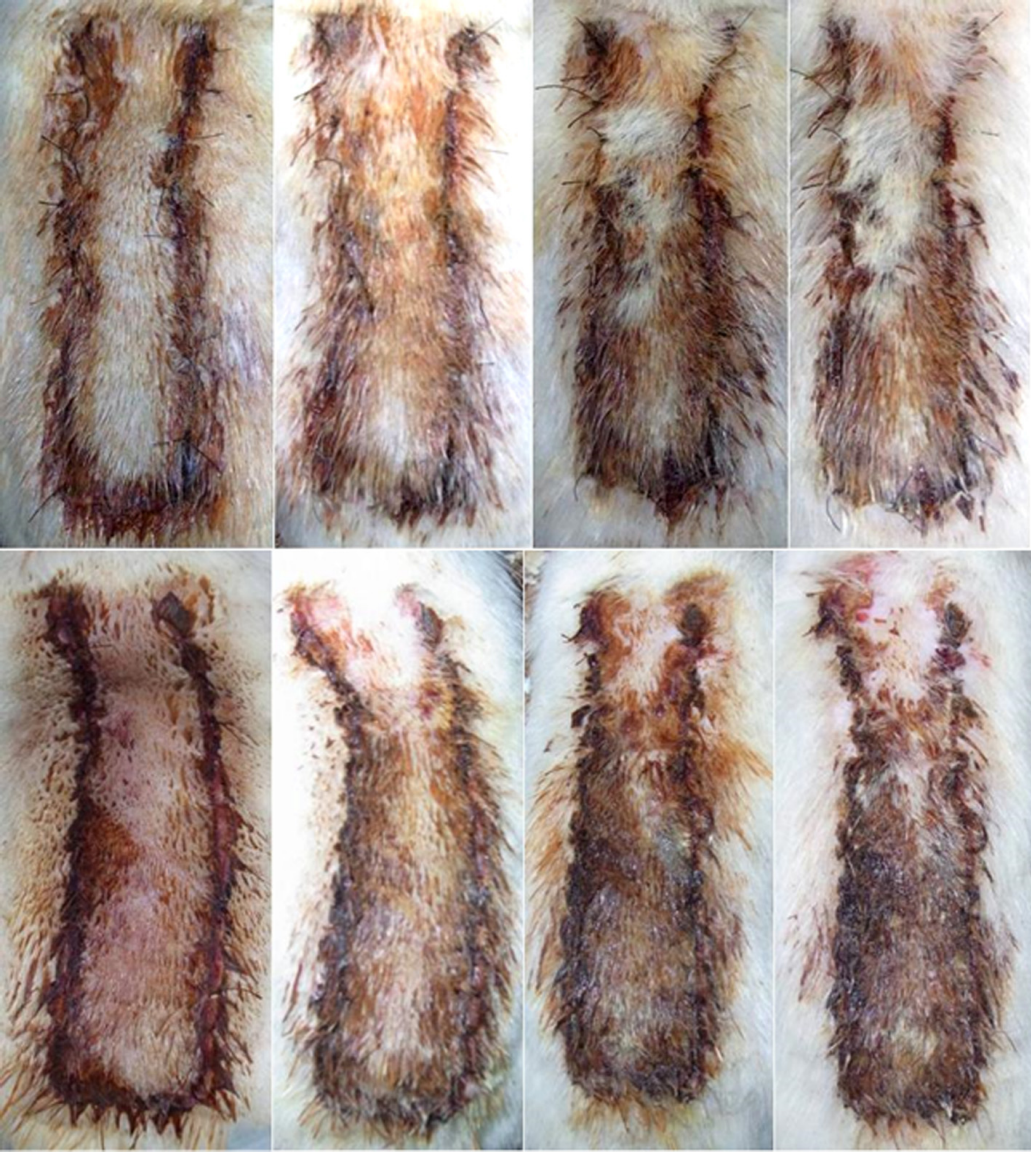
Comparison of flaps between cilostazol group (above) and control group (below) on postoperative day 1, 3, 5 and 7.

**Figure 3:**
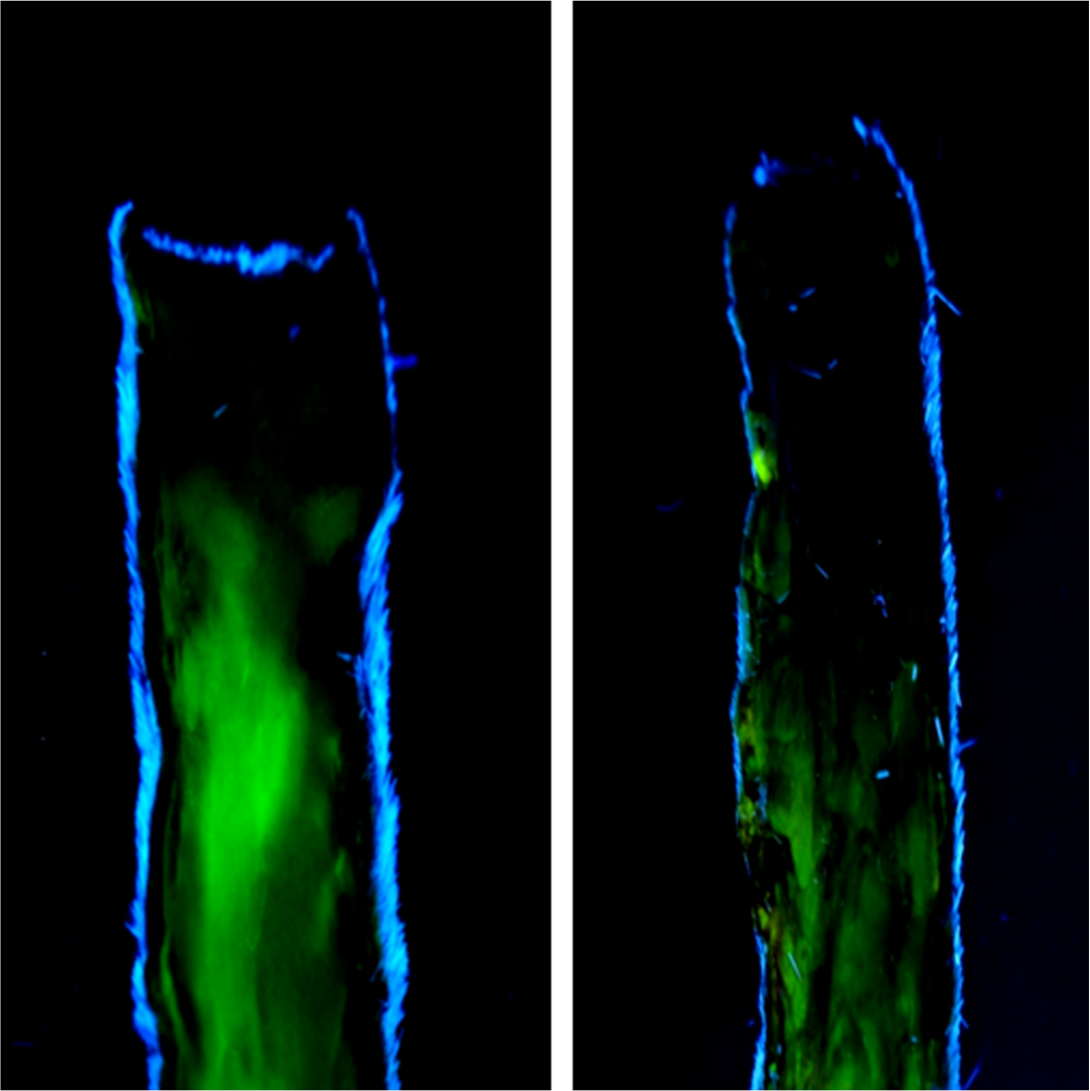
Fluorescein injection showed the higher area of flap survival in cilostazol group (left) than control group (right).

**Figure 4:**
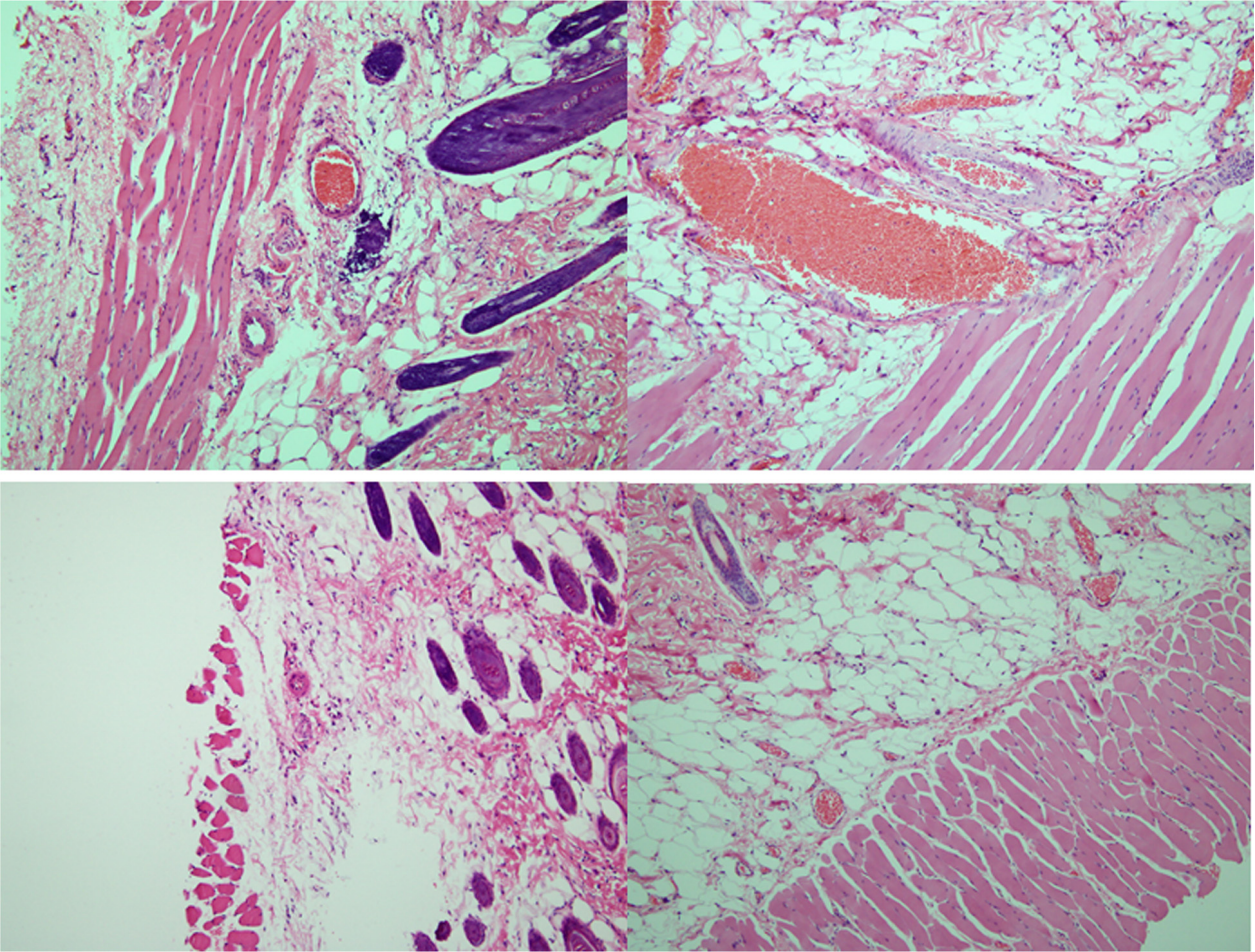
Histopathologic examination of the flap in cilostazol group (above) showed dilation of arterioles and venules. Compare with the control group (below), histopathologic examination showed a collapsed morphology of vessels (H&E stain X 100).

Infection and mortality were not observed during this study. Partial flap necrosis was observed on the distal part of the flap on day1,3,5 and 7 after flap elevation (Table 1) (Figure 2). The mean of flap survival on postoperative day 1 in the cilostazol group was 94.26% and in the control group was 91.17%. No statistical significance was found between each group (p = 0.064). The mean areas of flap survival in the cilostazol group were 80.3%, 52.69% and 49.76%, respectively and higher than the control group (75.41%, 42.9% and 40.08%) on post operative day 3, 5 and 7. The results between the groups administrated with pre-operative cilostazol and the control group were statistical significant on day 3, 5 and 7(p < 0.05). Fluorescein injection showed the higher area of flap survival in cilostazol group than control group (Figure 3). Statistical significance was found between cilostazol and control group when evaluation was performed by fluorescein injection (p < 0.05) (Table 2). No statistical significance was found between fluorescein injection and visual assessment on postoperative day 7. Histopathologic evaluation on postoperative day 7 showed dilatation of arterioles and venules in the cilostazol group. Compare with the control group, histopathologic examination showed a collapsed morphology of vessels (Figure 4).

**Table 1:** **Mean and standard deviation of the percentage of flap survival in each group**

**Day**	**Cilostazol (n = 15)**		**Control (n = 15)**		**p-value**
	**Mean(%) (min-max)**	**Standard deviation**	**Mean(%) (min-max)**	**Standard deviation**	
1	94.26 (89.01-100.00)	4.03	91.17 (85.34-100.00)	4.25	0.064
3	80.30 (70.65-85.67)	4.39	75.41 (69.98-81.67)	4.51	<0.05
5	52.69 (42.81-61.45)	4.87	42.90 (35.80-48.58)	3.72	<0.05
7	49.76 (40.89-58.67)	4.35	40.08 (32.78-45.67)	4.07	<0.05

**Table 2:** **The mean of the percentage of flap survival in each group, evaluation by fluorescein injection on postoperative day 7**

	**Cilostazol (n = 15)**	**Control (n = 15)**	**p-value**
	**Mean (%) (min-max)**	**Mean (%) (min-max)**	
Fluorescein injection	50.57 (41.56-60.12)	40.87 (33.77-46.35)	<0.05
Visual assessment	49.76 (40.89-58.67)	40.08 (32.78-45.67)	<0.05

## Discussion

Flap necrosis remains a significant problem in surgery. Flap necrosis is a result of simply inadequate end flow due to either vasoconstriction of the small arterioles or perfusion pressure decrease at a distance from the pedicle vessels. A random skin flap receives blood supply, mainly from subdermal plexus and distinguished from the axial flap that contains a dominant vessel inside. A random skin flap can be used without any specific problem as long as the ratio of length to width is around 1.5 – 2: 1 but if the ratio is higher, complications such as skin necrosis may occur when the flap is elevated [17].

Cilostazol is an inhibitor of phosphodiesterase type III, increases cyclic AMP (cAMP) in platelets and raises the vascular smooth muscle cell’s cAMP level causing arterial vasodilation. As an antithrombotic, it also reversibly inhibits platelet aggregation. Cilostazol has better properties than salicylic acid, such as a rapid onset (4 hours) and rapid cessation of the effect (within 48 hours) [18]. Although, side effects are diarrhea, headache, dizziness and contraindicated in patients with congestive heart failure, it is used to treat patients suffering from intermittent claudication without rest pain and no peripheral tissue necrosis as it improves pain-free walking distances [15,16].

Kim SH et al. [19] reported that cilostazol plus aspirin taken orally effectively increased the flow volume in a thrombotic anastomosis model in a rat. Yuzawa I [20] reported that oral administration of cilostazol can be used to decrease ischemic stroke. Nakamura et al. [21] reported that cilostazol induces vasodilation of the rat thoracic aorta and this effect was dependent on the endothelium, which released nitric oxide (NO) from aortic endothelial cell. Nitric oxide is an important role in flap physiology after flap elevation and final mediator to affect vascular smooth muscle causing arterial vasodilation. Many studies reported effect of cilostazol on nitric oxide production and showed that cilostazol potentiates interleukin-1 –beta (IL-1beta) induced NO production, at least partially through a cAMP-dependent pathway [22,23]. The cilostazol also might attenuate cytokine – induced expression of the inducible NO synthase protein expression(iNOS) and increase in the accumulation of nitrite, a stable met abolite of NO [23,24]. Some studies showed the advantage of locally application of cilostazol that inhibits neointimal hyperplasia of vein graft in rat model [25], increase blood flow in rabbit skin [26].Most of the previous studies reported the effect of cilostazol to improve blood flow, however, the effect of cilostazol on skin flap survival has not been established. In this study, cilostazol was used as a pharmacologic adjuvant therapy and the mechanism is considered to increase the survival of the flap. It is hypothesized that cilostazol may cause mainly on vascular smooth muscle and causing vasodilation, increasing blood volume to the flap either cAMP dependent pathway or NO production by increase iNOS and IL-1beta. A 2X8 cm^2^ cephalic based dorsal rat flap was advocated by McFarlane et al. in 1965 [27]. They originally described the dorsal skin flap of the rat as a random pattern flap for studying. In this study the authors used pre-operative at a dose of 40 mg/kg according to Nakamura et al. [21]. They reported that the serum concentration of cilostazol (40 mg/kg) in rats was about 1.5 ug/ml, equivalent to those of patients treated with cilostazol.

No statistical significance was found between each group on post operative day 1, maybe the area of flap necrosis on first day after the operation was difficult to determine. However, three days after operation, the demarcations of necrosis are well defined. Percentage of flap survival was statistical significant higher in cilostazol group than control group and confirmation of flap survival was performed by fluorescein injection and histopathologic evaluation.

Rat’s skin is different from human’s skin, especially, skin of rat has layer of panniculus carusus muscle. Therefore, a direct comparison between random skin flap of rat and human is difficult. Furthermore, the number of rats in each group was small, so further experiments in human with more subjects are needed.

## Conclusion

Pre-operative administration of cilostazol has significantly increased flap survival of a dorsal rat flap compared with the control group on post operative day 3,5 and 7. Herein, we hoped that this study will result in better treatments and result guidelines to be benefit of patients in the future.
